# Arsenic, cadmium and neuron specific enolase (ENO2, γ-enolase) expression in breast cancer

**DOI:** 10.1186/1475-2867-11-41

**Published:** 2011-11-18

**Authors:** Maureen A Soh, Scott H Garrett, Seema Somji, Jane R Dunlevy, Xu Dong Zhou, Mary Ann Sens, Chandra S Bathula, Christina Allen, Donald A Sens

**Affiliations:** 1 Department of Pathology, School of Medicine and Health Sciences, University of North Dakota, Grand Forks, ND, USA; 2 Department of Anatomy and Cell Biology, School of Medicine and Health Sciences, University of North Dakota, Grand Forks, ND, USA

**Keywords:** Biomarker, arsenic, cadmium, breast cancer, breast epithelial cells, MCF-10A, enolase, ENO, neuron specific enolase, ENO2

## Abstract

**Background:**

Neuron specific enolase (ENO2, γ-enolase) has been used as a biomarker to help identify neuroendocrine differentiation in breast cancer. The goal of the present study was to determine if ENO2 expression in the breast epithelial cell is influenced by the environmental pollutants, arsenite and cadmium. Acute and chronic exposure of MCF-10A cells to As^+3 ^and Cd^+2 ^sufficient to allow colony formation in soft agar, was used to determine if ENO2 expression was altered by these pollutants.

**Results:**

It was shown that both As^+3 ^and Cd^+2 ^exposure caused significant increases in ENO2 expression under conditions of both acute and chronic exposure. In contrast, ENO1, the major glycolytic enolase in non-muscle and neuronal cells, was largely unaffected by exposure to either As^+3 ^or Cd^+2^. Localization studies showed that ENO2 in the MCF-10A cells transformed by As^+3 ^or Cd^+2 ^had both a cytoplasmic and nuclear localization. In contrast, ENO1 was localized to the cytoplasm. ENO2 localized to the cytoplasm was found to co-localized with ENO1.

**Conclusion:**

The results are the first to show that ENO2 expression in breast epithelial cells is induced by acute and chronic exposure to As^+3 ^or Cd^+2^. The findings also suggest a possible link between As^+3 ^and Cd^+2 ^exposure and neuroendocrine differentiation in tumors. Overall, the results suggest that ENO2 might be developed as a biomarker indicating acute and/or chronic environmental exposure of the breast epithelial cell to As^+3 ^and Cd^+2^.

## Background

This laboratory is interested in the identification of biomarkers that indicate potential human exposure to environmental agents. Historically, neuron specific enolase was one of three immunohistochemical markers that were used to determine tumors that possessed a subpopulation of tumor cells with neuroendocrine differentiation, the other markers being chromogranin A and synaptophysin [1]. Enolase is an enzyme of the glycolytic pathway that catalyzes the conversion of 2-phosphoglycerate into phosphoenolpyruvate [2]. In mammalian tissues, enolase has three tissue-specific isoenzymes [3-5]. Enolase molecules are dimers composed of three distinct subunits coded by separate genes and originally designed α (liver), β (muscle) and γ (brain). The αα isoenzyme is present in all fetal tissues and most adult mammalian tissues. The ββ- and γβ-enolase are found predominantly in skeletal and heart muscle. The γγ- and αγ-enolase are present mainly in nervous tissue and in tissues with neuroendocrine cells. The γγ- and αγ-enolase have been frequently referred to as neuron-specific enolase (NSE), especially when used as a marker to aid cancer diagnosis and characterization. The distribution of the enolase isoenzymes has been determined in normal and malignant human breast by electrophoresis [6]. In normal human breast tissue the αα-enolase is the predominant enolase isoenzyme. The αγ- enolase is present in much lower amounts (2-16%), and the levels of γγ-enolase are very low (0 - 3%). A corresponding analysis of breast carcinomas shows a distribution of enolase isoenzymes similar to that of normal breast, but with the proportion of the γ-possessing isoenzymes being higher than normal tissue. Using immunohistochemistry with an antibody against γ-enolase, no positive staining for γ-enolase was found in several studies employing normal breast tissue [7-10]. In contrast, a high proportion of γ-enolase staining cells was detected in some breast carcinomas [5,7,8,10-16]. The designations ENO1, ENO2, and ENO3 is also commonly used in the literature to designate the α, γ and β enolase genes and gene products, respectively.

The extensive studies of neuron-specific enolase expression in normal breast and breast carcinomas suggested that the breast epithelial cell would be an appropriate model on which to determine if arsenite and cadmium might alter the expression of neuron-specific enolase. The first goal of the present study was to determine if acute exposure of MCF-10A cells to As^+3 ^or Cd^+2 ^altered the expression of the ENO1 and ENO2 genes. The second goal of the study was to determine if chronic exposure to As^+3 ^or Cd^+2^, sufficient to elicit cell transformation as judged by colony formation in soft agar, would alter the expression of ENO1 and ENO2.

## Results

### Immunohistochemical staining of ENO1, ENO2 and ENO3 in normal human breast

**Figure 1:**
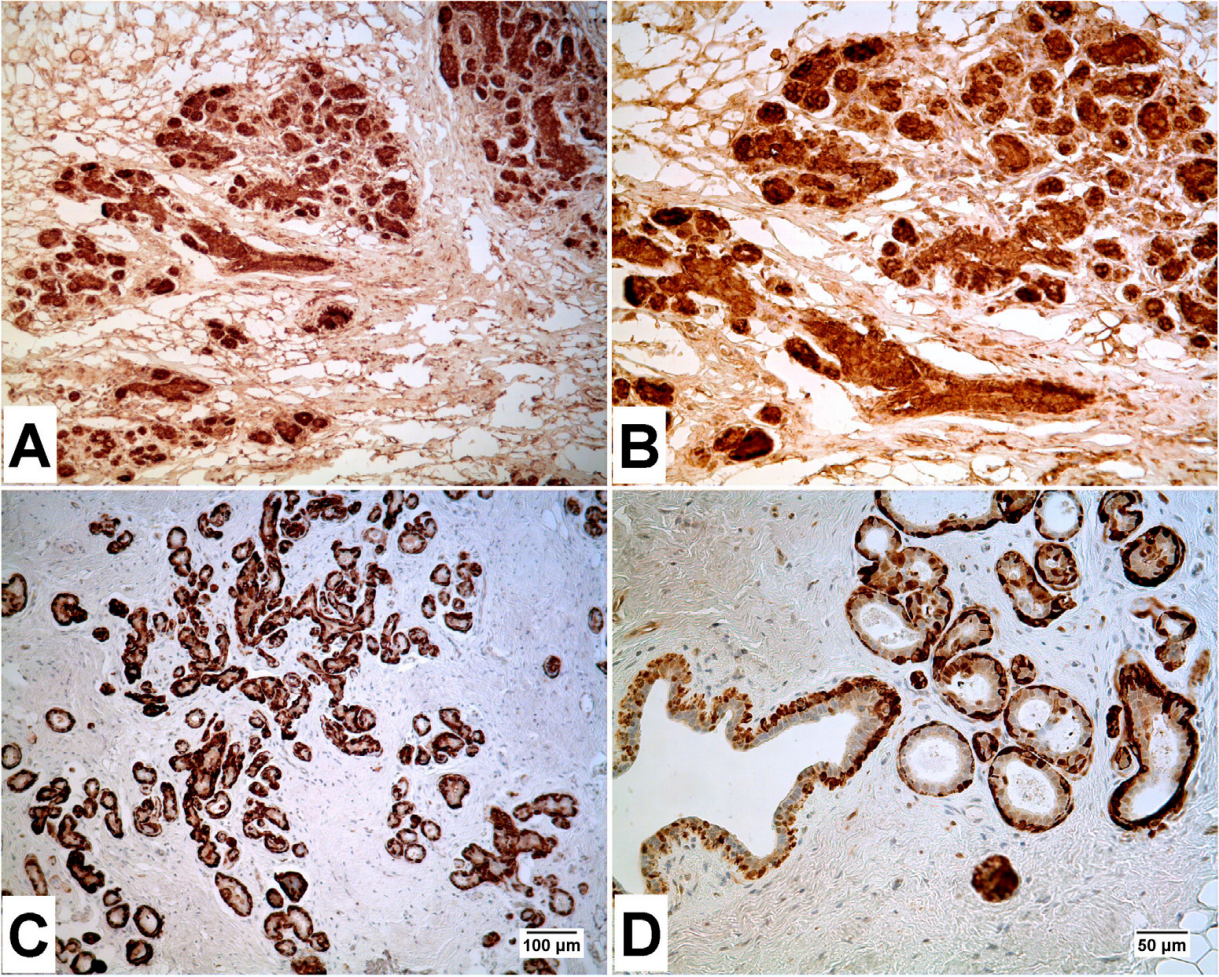
**Immunohistochemistry of ENO1, ENO2 and ENO3 in Normal Human Breast**. Formalin-fixed, paraffin-embedded human breast biopsy specimens were immunohistochemically stained with specific antibodies to ENO1 (A and B) or ENO2 (C and D). Color development was performed via an HRP-linked secondary antibody with diaminobenzidine as the enzyme substrate, giving light to dark brown color in regions exhibiting ENO protein expression. ENO1 shows staining in both myoepithelial and epithelial cells of the breast lobules and ducts whereas ENO2 shows expression mainly in the myoepithelial cells with a slight staining in the epithelial cells of breast lobules and ducts. Magnification 100× for A and C; 200× for B and D.

The immunohistochemical staining of ENO1, 2 and 3 was determined on 3 archival samples of formalin-fixed, paraffin-embedded normal human breast tissue. The staining pattern was similar among all three independent tissue samples. Intense staining for ENO1 was localized to the ductal epithelial and myoepithelial cells of the breast (Figure 1A, B). The stromal elements of the breast had moderate to weak staining for the expression of ENO1. The staining for ENO2 was strong in the outer myoepithelial cells of the breast ducts (Figure 1C, D). The stromal elements of the breast were negative for the expression of ENO2. The ductal epithelial elements of the breast, when compared to the stroma, were at or slightly above, background levels of staining. There was no staining of ENO3 in any cells of the normal breast (data not shown).

### ENO1, 2 and 3 expression in the MCF-10A cell line

**Figure 2:**
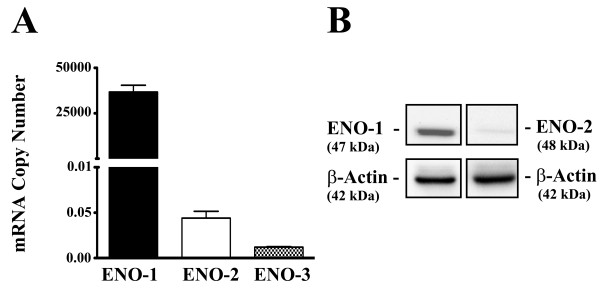
**Expression of ENO-1, -2 and -3 mRNA and protein in MCF-10A parent cell line**. Real-time PCR analysis of ENO-1, -2 and -3 transcript copy number in the MCF-10A parent cell line (A) and western blot analysis of ENO-1 and -2 in the MCF-10A parent cell line (B). Transcript copy number was assessed using quantitative DNA standards and the levels shown are for an RNA input of 40 ng.

The expression of ENO1, 2 and 3 mRNA and ENO1 and 2 protein were determined on total RNA and protein prepared from confluent cultures of the MCF-10A cell line. The expression of ENO1 mRNA was detected in MCF-10A cells at levels similar to that of β-actin mRNA (Figure 2A). In contrast, the expression of both ENO2 and ENO3 mRNA was very low and over 100 fold less when compared to that of ENO1 mRNA in the MCF-10A cell line (Figure 2A). Western analysis showed that the MCF-10A cells expressed the ENO1 protein at levels in a large excess to those found for ENO2 and ENO3. The ENO2 protein was just visible on western analysis (Figure 2B). The levels of the ENO3 protein were not determined since there was no evidence of ENO3 mRNA.

### ENO 1, ENO2 and ENO3 expression in MCF-10A cells acutely exposed to Cd^+2 ^or As^+3^

**Figure 3:**
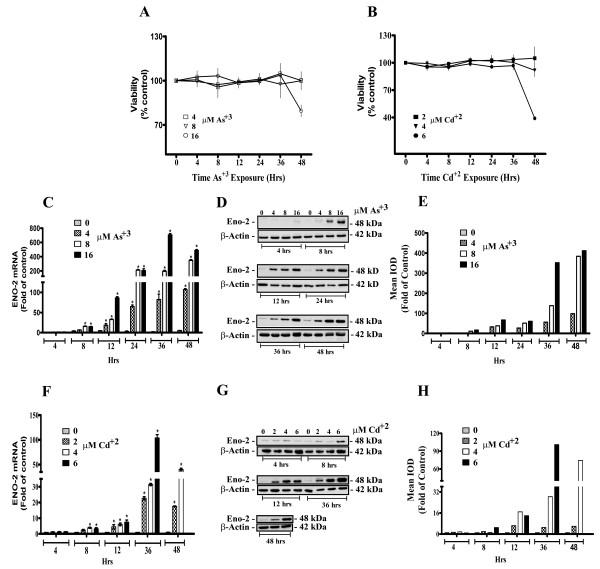
**Expression of ENO-2 mRNA and protein in parental MCF-10A cells exposed to As^+3 ^and Cd^+2^**. Cell viability, as an indicator of cytotoxicity, was determined by measuring the capacity of the MCF-10 cells to reduce MTT (3-(4, 5-dimethylthiazol-2-yl)-2, 5-diphenyltetrazolium bromide) to formazan after exposure to As^+3 ^(A) and Cd^+2 ^(B). Real-time RT-PCR analysis of ENO-2 mRNA in parental MCF-10A cells exposed to As^+3 ^and Cd^+2 ^(C and F). Western blot analysis of ENO-2 protein in parental MCF-10A cells exposed to As^+3 ^and Cd^+2 ^(D and G) and western blot densitometric analysis (E and H) respectively. Statistical analysis consisted of ANOVA with Tukey post hoc testing. *Denotes a statistically significant difference from untreated MCF-10A parent cells (p < 0.05). Real-time data is plotted as the mean ± SEM of triplicate determinations.

The expression of ENO2 mRNA and protein were determined in the MCF-10A cells exposed to Cd^+2 ^and As^+3 ^for 48 hrs. Three concentrations of As^+3 ^(4, 8 and 16 μM) and Cd^+2 ^(2, 4 and 6 μM) were used and were chosen such that the highest level showed a loss of cell viability following 48 hrs of exposure (Figure 3A, B). The results showed that both acute exposure to either As^+3 ^or Cd^+2 ^increased the expression of ENO2 in MCF-10A cells (Figure 2C-H). Exposure of the MCF-10A cells to the lowest level of As^+3 ^(4 μM) caused a significant increase in ENO2 mRNA expression following 12 hrs of exposure (Figure 3C). The two higher concentrations of As^+3 ^(8 and 16 μM) elicited a significant increase in ENO2 mRNA expression following 12 hours of exposure (Figure 3C). Western analysis for ENO2 protein expression confirmed the increases in ENO2 mRNA expression (Figure 3D, E). The ENO2 protein expression could be noted on the western blots at time points when ENO2 mRNA was just beginning to be elevated following exposure of the MCF-10A cells to As^+3^. Exposure of the MCF-10A cells to the lowest level of Cd^+2 ^(2 μM) resulted in a significant increase in ENO2 mRNA following 12 hrs of exposure, while the two higher dosages (4 and 6 μM) caused significant increases following 8 hrs of exposure (Figure 3F). The expression of the ENO2 protein was clearly elevated by 12 hrs of exposure to all three concentrations of Cd^+2 ^(3 G, H). When judged as matched for similar toxicity as shown by the viability curves, exposure to As^+3 ^appeared to be a stronger inducer of ENO2 expression.

**Figure 4:**
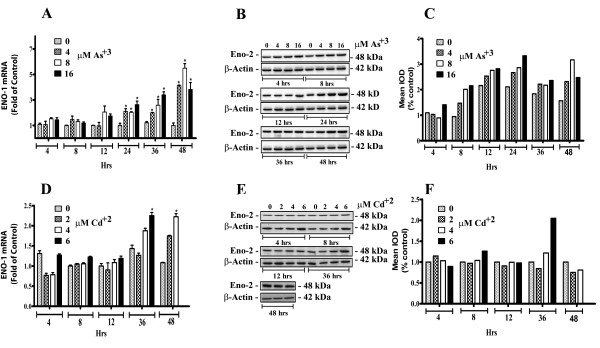
**Expression of ENO-1 mRNA and protein in parental MCF-10A cells exposed to As^+3 ^and Cd^+2^**. Real-time RT-PCR analysis of ENO-1 mRNA in parental MCF-10A cells exposed to As^+3 ^and Cd^+2 ^(A and D). Western blot analysis of ENO-1 protein in parental MCF-10A cells exposed to As^+3 ^and Cd^+2 ^(B and E) and western blot densitometric analysis (C and F) respectively. Statistical analysis consisted of ANOVA with Tukey post hoc testing. *Denotes a statistically significant difference from untreated MCF-10A parent cells (p < 0.05). Real-time data is plotted as the mean ± SEM of triplicate determinations.

The expression of ENO1 mRNA and protein was determined on identical samples to those described above. The results showed that for the MCF-10A cells exposed to As^+3 ^that there were significant increases in ENO1 mRNA following 24 hrs of exposure for all three As^+3 ^concentrations (Figure 4A). However, these increases were modest in comparison to those noted for ENO2 mRNA under identical conditions of exposure. The increase in ENO1 mRNA expression was accompanied by a trend for increased ENO1 protein expression but the increases in ENO1 protein were not significant compared to control (Figure 4B, C). The results showed that for MCF-10A cells exposed to Cd^+2 ^that there was no significant increase in ENO1 mRNA for the 2 lower Cd^+2 ^concentrations and at only two time points for the highest Cd^+2 ^concentration (Figure 4D). There were no large increases in ENO1 protein at any of the three Cd^+2 ^concentrations with the exception of 6 μM at 36 hr (Figure 4E, F). There was no expression above background for ENO3 mRNA for the MCF-10A cells exposed to As^+3 ^or Cd^+2 ^(data not shown) and for this reason ENO3 protein was not determined on the samples.

### Transformation of MCF-10A cells with As^+3 ^and Cd^+2^

**Figure 5:**
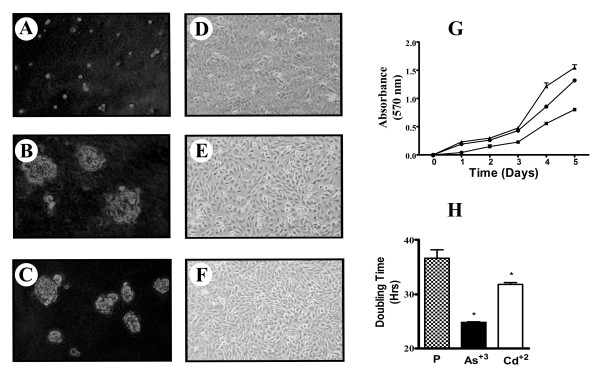
**Soft agar and light microscopic morphology of MCF-10A parent cells, before and after malignant transformation**. Soft agar and light microscopic morphology of control MCF-10A cells (A and D), As^+3^- transformed MCF-10A cells (B and E) and Cd^+2^- transformed MCF-10A cells (C and F) respectively. Growth rates and doubling times of control MCF-10A cells and MCF-10A cells after malignant transformation with As^+3 ^or Cd^+2 ^(G and H) respectively. Soft agar and light microscopy pictures taken at 200 × and 100 × magnifications respectively.

The transformation of MCF-10A cells with As^+3 ^and Cd^+2 ^followed a protocol very similar to that described previously by this laboratory for the malignant transformation of single cultures of UROtsa cells, an immortalized culture of urothelial cells [17-19]. The MCF-10A cells were exposed to 1 μM As^+3 ^or Cd^+2 ^and each were monitored by light microscopy 24 hrs before and after each change of the growth medium and subcultured when the cells reached confluence. The first assessment of the cells ability to form colonies in soft agar was performed following the first four serial passages and thereafter every 4 to 5 serial passages. Cells were also preserved in liquid nitrogen at the time of assessment for growth in soft agar. At serial passage 25 both sets of the As^+3 ^and Cd^+2 ^transformed cells were able to form colonies in soft agar (Figure 5A, B, C). At this passage number, multiple flasks of cells were removed from liquid nitrogen storage and multiple vials processed for long-term storage under liquid nitrogen. The light level morphology of the As^+3 ^and Cd^+2 ^transformed cell lines able to grow in soft agar were very similar to that of parental MCF-10A cells (Figure 5D, E, F). There was no evidence at the light level of examination that the cells had lost the contact inhibition of growth or that they formed multilayered foci of cells. The doubling time of both the As^+3 ^and Cd^+2 ^transformed cells did decrease significantly compared to control MCF-10A cells (24.9 ± 0.1, 32.2 ± 0.3, and 39.7 ± 1.6 hrs, respectively).

The As^+3 ^and Cd^+2 ^transformed cell lines having the ability to form colonies in soft agar were each inoculated subcutaneously at a dose of 1 × 10^6 ^cells at the dorsal thoracic midline of 5 nude mice to determine if the cell lines able to form colonies in soft agar were also capable of forming tumors in immune compromised mice. The mice were assessed every two weeks for tumor formation and following 9 months of examination there was no evidence of tumor formation as noted by the growth of nodules under the skin. The mice were sacrificed at 9 months and the injection site under the skin surface examined visually for any evidence of tumor nodules. In no instance was any evidence of tumor formation found in any of the mice. Extending the serial passages of the MCF-10A cells in both the presence and absence of As^+3 ^and Cd^+2 ^for an additional 15 passages also had no effect on tumor formation.

### Expression and localization of ENO1 and ENO2 in MCF-10A cells transformed by As^+3 ^and Cd^+2^

**Figure 6:**
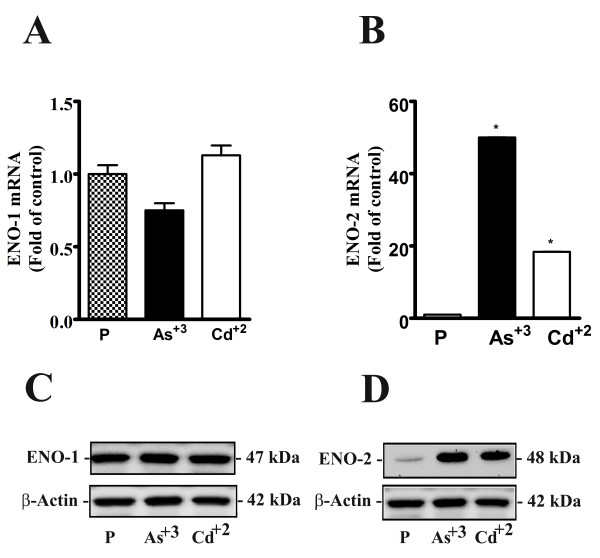
**Expression of ENO-1 and ENO-2 mRNA and protein in As^+3 ^and Cd^+2^- transformed MCF-10A cells**. Real-time RT-PCR and western blot analysis of ENO-1 mRNA and protein in As^+3 ^and Cd^+2^- transformed MCF-10A cells (A and C) and ENO-2 (B and D) respectively. Statistical analysis consisted of ANOVA with Tukey post hoc testing performed using GraphPad PRISM 5. *Denotes a statistically significant difference from MCF-10A parent cells (p < 0.05). Real-time data is plotted as the mean ± SEM of triplicate determinations.

The expression of ENO1 and ENO2 mRNA and protein were determined on the MCF-10A cell line transformed by As^+3 ^and Cd^+2 ^(Figure 6A-D). The results showed that the expression of ENO1 mRNA and protein by the MCF-10A cells transformed with either As^+3 ^or Cd^+2 ^was not altered from that of the parental MCF-10A cell line (Figure 6A, C). In contrast, it was shown the ENO2 mRNA and protein were increased significantly by the MCF-10A cells transformed by either As^+3 ^or Cd^+2 ^when compared to the parental MCF-10A cells (Figure 6B, D).

**Figure 7:**
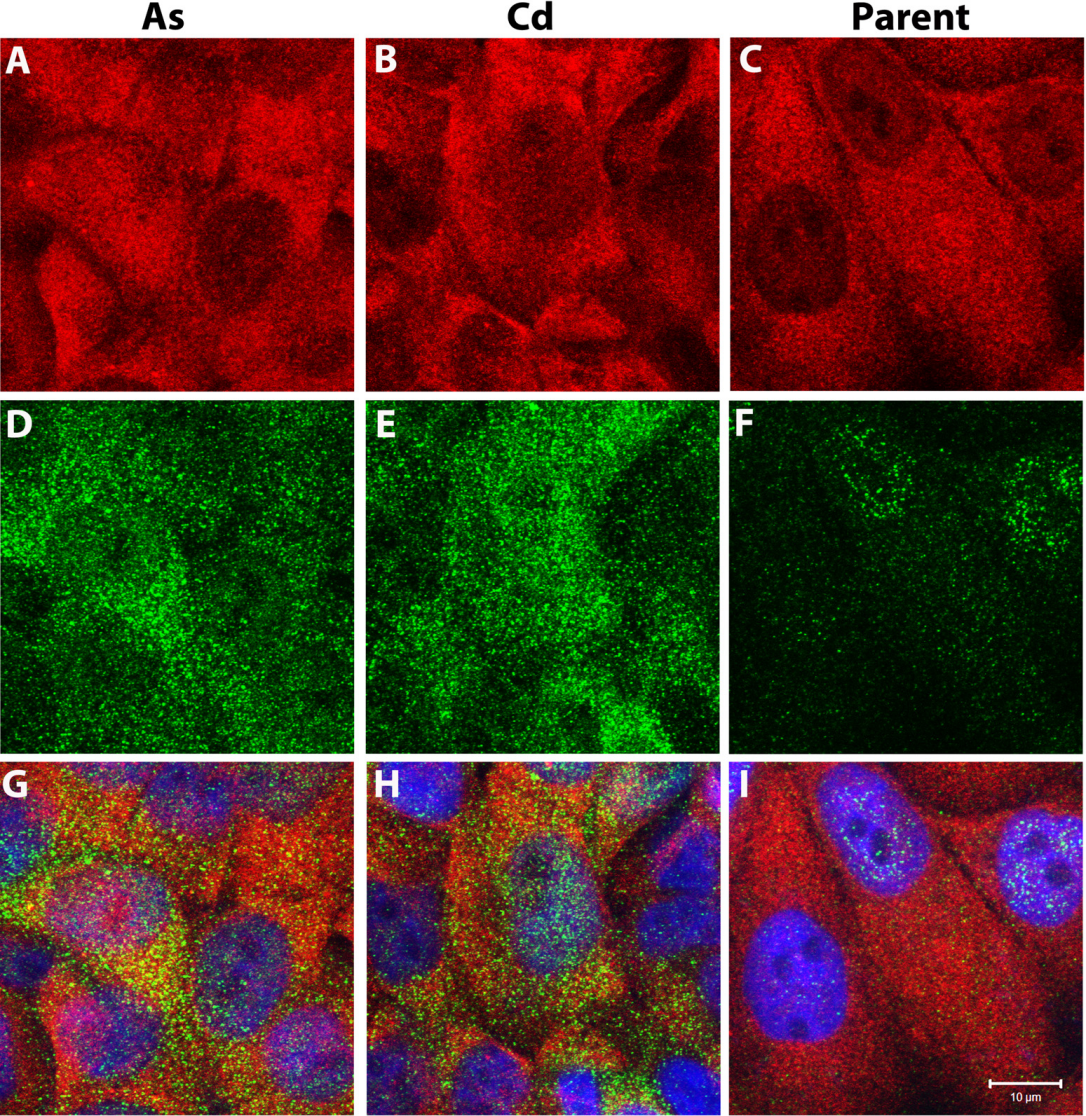
**Localization of enolase-1 and -2 in the MCF-10A cell lines**. Cells were stained for enolase-1 (red) shown in A-C and for enolase-2 (green) shown in D-F. These confocal images were taken through the z-series and then the z-plane images were stacked together to form the image shown. Therefore, each image represents the total cellular enolae-1 or -2 staining within each field. Subsequently, the enolase-1 and -2 images were then merged together along with the epifluorescent image of the DAPI nuclear stain (blue) and are shown in G-I. The field of MCF-10A As^+3 ^transformed cells are shown in A, D, and G: the field of MCF-10A Cd^+2 ^transformed cells are shown in B, E, and H; and the non-transformed MCF-10A parent cells are shown in C, F, and I. Bar = 10 μm.

Immunofluorescence was used to localize the expression of ENO1 and ENO2 in the MCF-10A parental cells and their As^+3 ^and Cd^+2 ^transformed counterparts. In all three cell lines, the intracellular localization of ENO1 was diffuse within the cytoplasm with only a very minor fraction of total cellular ENO1 localized to the nuclear region (Figure 7A-C). This pattern was found consistently throughout the cell population and there were no differences observed in ENO1 staining intensity or intracellular distribution among the three cell lines. In all three cell lines, the intracellular localization of ENO2 was characterized by both diffuse, punctate cytoplasmic staining and by substantial levels of staining localizing to the nuclear region (Figure 7D-F). All three cell lines displayed heterogeneous levels of ENO2 staining intensity within the cell population. In contrast to the transformed cell lines, many profiles of parental MCF-10A cells were either negative for the expression of ENO2 or had low levels, particularly within the cytoplasm. Visual observation of fluorescence intensity also suggested that the As^+3 ^and Cd^+2 ^transformed cell lines have higher levels of ENO2 compared to parental cells. This difference is very evident in the merged images of ENO1 and ENO2 staining (Figure 7G-I). These images also show an overlap within the cytoplasm of ENO1 and ENO2 localization as well as an overlap of predominately ENO2 with the nucleus.

**Figure 8:**
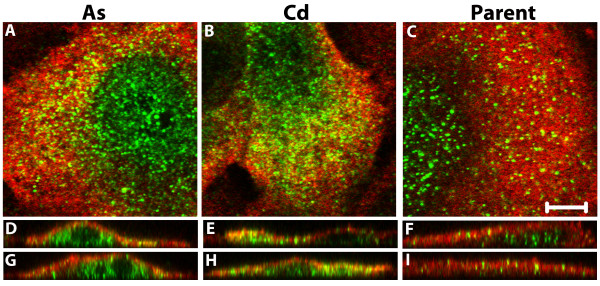
**Co-localization for enolase-1 and -2**. Cells were stained for enolase-1 (red) and then for enolase-2 (green). Co-localization is shown in the cytoplasm (yellow) for the As^+3 ^transformed (A, D, G), Cd^+2 ^transformed (B, E, H) and parent MCF-1-A (C, F, I) cells. Single z-series slices of 0.46 μm are shown in A-C with orthogonal slices of the z-series shown for the x-axis in D-F and the y-axis in G-I. The bar indicates the scale for images in A-C; bar = 5 μm.

The co-localization of ENO1 and ENO2 was also determined among the cell lines by capturing optimal z-series confocal slices of 0.46 μm. These images were examined as single slices (Figure 8A-C) as well as orthogonal views of the z-series through the x- and y-planes (Figure 8D-I). Co-localization of ENO1 and ENO2 was demonstrated in the cytoplasm of cells from all three cell lines. There were also instances of cells in all three cell lines that were observed to have little to no co-localization of ENO1 and ENO2. However, co-localization of ENO1 and ENO2 was observed in a high proportion of fields of all captured cells. Co-localization appeared to be dependent on the levels of ENO2, with cells having higher levels of cytoplasmic ENO2 being more likely to show co-localization with ENO1. When comparing the As^+3 ^and Cd^+2 ^transformed cells to the parent MCF-10A cells, ENO1 and ENO2 were observed to not only co-localize in a higher number of cells within the population of the transformed cell lines but to also have more co-localization within individual cells than that of the parent cell line. This difference is most likely simply due to there being substantially more ENO2 in the As^+3 ^and Cd^+2 ^transformed cell lines. Possible co-localization within the nucleus could not be determined due to the very low levels of ENO1 in the nucleus.

**Figure 9:**
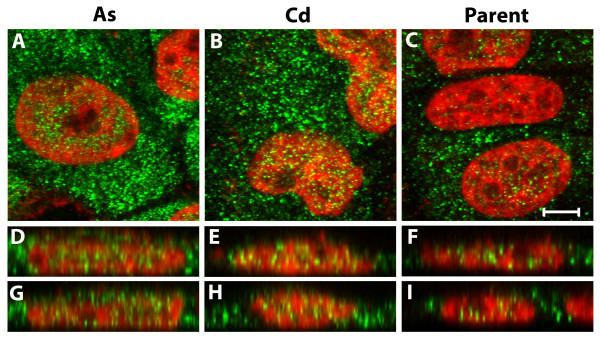
**Localization of enolase-2 to the nucleus**. Cells were stained for enolase-2 (green) followed by staining for the nuclei with To-PRO-3 iodide (red). Localization within the nucleus is shown for the As^+3 ^transformed (A, D, G), Cd^+2 ^transformed (B, E, H) and parent MCF-10A (C, F, I) cells. Single z-series slices of 0.46 μm are shown in A-C with orthogonal slices of the z-series shown for the x-axis in D-F and the y-axis in G-I. The bar indicates the scale for images in A-C; bar = 5 μm.

The images in the above Figures provide evidence that ENO2 can be localized in the cell nucleus. Further evidence was obtained by staining for ENO2 followed by treating the cells with To-PRO-3 iodide. Optimal z-series confocal slices of 0.46 μm were captured and examined as both single slices as well as orthogonal views of the z-series through the x- and y-planes (Figure 9). The results showed that ENO2 staining was closely associated with the nucleus (Figure 9A-C). In the orthogonal views, results show ENO2 staining within the nucleus structure itself (Figure 9D-I). The ENO staining also tended to cluster in regions of the nucleus that had low To-PRO-3 iodide staining. This may indicate that ENO2 associates with euchromatin versus the more intensely staining heterochromatin.

### ENO2 mRNA expression in parental MCF-10A cells following treatment with inhibitors of DNA methylation and acetylation

**Figure 10:**
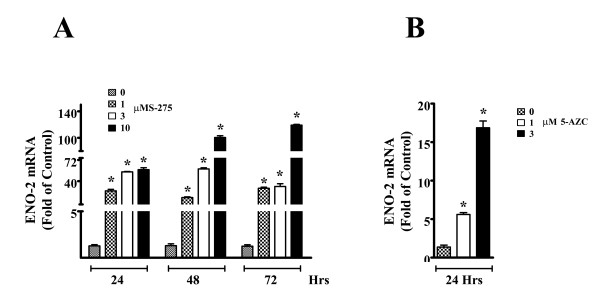
**Real-time RT-PCR analysis of ENO-2 mRNA levels in parental MCF-10A cells treated with epigenetic regulators**. Figure 10 (A) shows expression of ENO-2 mRNA after treatment with histone deacetylase Inhibitor, MS-275 for up to 72 hrs and (B) shows expression of ENO-2 mRNA after treatment with the DNA demethylation agent, 5-AZC, for 24 hrs. Statistical analysis consisted of ANOVA with Tukey post hoc testing performed using GraphPad PRISM 5. *Denotes a statistically significant difference from untreated MCF-10A parent cells (p < 0.05). Real-time data is plotted as the mean ± SEM of triplicate determinations.

The MCF-10A parental cell line was treated with the histone deacetylase inhibitor, MS-275, and the methylation inhibitor, 5-AZC, to determine the possible role of epigenetic modifications on ENO2 mRNA expression. This analysis demonstrated that treatment of the cells with either MS-275 or 5-AZC significantly increased the expression of ENO2 mRNA compared to control cells (Figure 10A, B). The samples utilized were generated in a previous study and the concentrations and times used resulted in no cell death [20].

## Discussion

The expression and localization of ENO1, ENO2 and ENO3 was determined on three archival samples of normal human breast tissue to determine if the antibody used in the present study produced results in agreement with past reports in the literature. Immunostaining for ENO1 showed all the cells of the normal breast to have expression of ENO1. This is in agreement with the literature which reports the ubiquitous expression of ENO1 in adult tissues and cells [6,21,22]. Immunostaining for ENO2 showed the myoepithelial cells of the normal breast to have strong staining for ENO2. This pattern of expression is in agreement with an earlier study on ENO2 expression in normal human tissues other than the nervous system [5]. This finding is also in agreement with the finding of ENO1/ENO2 and ENO2 isoenzymes in extracts of human breast tissue that were analyzed using electrophoresis [6]. It is very possible that the expression of ENO2 found in this past report was from the myoepithelial cells of the breast and not from the ductal epithelial cells. However, the present immunostaining studies of ENO2 in the normal breast could not rule out a very low expression of ENO2 at or near background levels in the breast epithelial cells. Consistent with the literature, immunostaining showed no expression above background for ENO3 in the normal human breast [6]. The analysis of ENO1, 2 and 3 mRNA and protein expression in the MCF-10A cell line was in agreement with the immunostaining results found in the normal breast. This study showed that the MCF-10A cell line had a high level of ENO1 expression in both mRNA and protein. ENO2, however, was expressed at a considerably lower level with western analysis barely detecting the protein on gels. Analysis if ENO3 mRNA expression was near the detection limit of the PCR assay, and the protein expression was not analyzed further due to the absence of appreciable mRNA expression. Immunofluorescent localization showed all cells to expression ENO1 protein and a much lower percentage of cells to express ENO2 protein and when present, a much lower intensity of expression. Overall, these findings confirmed that the MCF-10A cells modeled the expression of ENO1, 2 and 3 similar to that found in the epithelial cells of the normal breast.

To the authors knowledge there has been no examination of the effect that exposure to Cd^+2 ^or As^+3 ^has on the expression of enolase in the breast epithelial cell or other epithelial cell types. The enolases do have a metal requirement for activity, and six divalent metal ions, magnesium, manganese, zinc, cadmium, cobalt and nickel, can activate enolase [21]. However, these metals are required for enzyme activity and none of the six divalent metals has been shown to increase the expression of any of the enolases at the level of mRNA or protein. The demonstration in the present study that acute exposure to either As^+3 ^or Cd^+2 ^increases the expression of ENO2 mRNA and protein is the first to suggest a role of these metals in the elevation of neuron specific enolase in the breast epithelial cell and possibly other epithelial cell types. That the induction of ENO2 by As^+3 ^and Cd^+2 ^is specific for the acute induction of ENO2 expression is strongly suggested by the finding that identical exposure resulted in only a limited induction of ENO1 mRNA and no induction of ENO1 protein and no increase in ENO3 mRNA. These findings suggest that ENO2 expression might be translated as a possible biomarker for acute environmental exposure to either As^+3 ^or Cd^+2^.

The properties of ENO1 have been studied in far greater detail than that of ENO2, even though ENO2 has been employed extensively as a tumor marker for neuroendocrine differentiation. The finding that ENO2 is specifically induced by As^+3 ^and Cd^+2 ^in the breast epithelial cell calls into question how much of the accumulated knowledge known for ENO1 can be translated to the ENO2 isoform. It is known that ENO1 is a multifunctional protein and has functions beyond that of its central role in glycolysis [21]. ENO1 is expressed on the surface of a variety of eukaryotic cells as a strong plasminogen-binding receptor. This has been shown for hematopoietic cells, epithelial cells, and endothelial cells [21,22]. The mechanism by which ENO1 reaches the cell surface is unknown. In addition, ENO1 has also been shown to have an alternative protein form known as the Myc-binding protein, MBP-1 [21,23]. The start codon for MBP-1 ATG resides 400 bp downstream of the ENO1 ATG and thus is considered to be an alternative translation initiation product of ENO1 mRNA. In endothelial cells, ENO1 has been shown to be a hypoxic stress protein. The possible role of ENO1 in various disease states has also received far greater study than that of ENO2 [21]. Many of these possible roles are related to the surface expression of ENO1 and plasminogen binding since this may play an important role in modulating the pericellular and intravascular fibrinolytic system. It does not appear that similar studies have been undertaken for the ENO2 isoform, and it is unknown if the properties known for ENO1 protein are also present for ENO2. The only caveat is that this laboratory's analysis of the ENO2 mRNA sequence showed no evidence of an alternative initiation site that could specifically encode the MBP-1 Myc-binding protein.

The MCF-10A cell line was transformed by exposure to As^+3 ^and Cd^+2 ^to determine if this would result in altered expression of ENO2 mRNA and protein. Previous studies have shown that the MCF-10A cell line can be transformed by exposure to Cd^+2 ^[24], but this has not been shown previously for As^+3^. In the present study, transformation was judged to have occurred when the As^+3 ^and Cd^+2 ^exposed MCF-10A cells were able to produce colonies in soft agar. The ability for colony formation in soft agar is typical of cancer cells and is thought to reflect anchorage-independent growth of tumor-initiating/cancer stem cells [25,26]. The As^+3 ^and Cd^+2 ^transformed cell lines were unable to form tumors when inoculated subcutaneously in immune compromised mice. This may be due to the site of inoculation, since the earlier study showing the transformation of MCF-10A cells with Cd^+2^, the cells were injected under the renal capsule [24], a technique not available at this laboratory's animal facility. The current study showed that the expression of ENO2 was significantly elevated in both As^+3 ^and Cd^+2 ^transformed cell lines compared to parent MCF-10A cells with the As^+3^-transformed cells having higher expression than that of the Cd^+2^-transformed cells. ENO1 expression was not altered in these cells. Similar to that found for acute exposure, these findings suggest that ENO2 expression might be translated as a possible biomarker for chronic environmental exposure to either As^+3 ^or Cd^+2^. The elevated expression of ENO2 in the As^+3 ^and Cd^+2 ^transformed cell lines allowed an analysis of the intracellular localization of ENO2 as well as its interaction with ENO1. It was shown in these cell lines that ENO1 has a diffuse, mainly cytoplasmic localization while ENO2 displays both a punctate, cytoplasmic localization and a nuclear localization. The cytoplasmic ENO1 and ENO2 were shown to co-localize. There was no evidence of any nuclear ENO1 localization or any ENO1 co-localization with nuclear ENO2. There does not appear to be previous literature reports of nuclear ENO2 localization in human epithelial cancer cells. ENO2 has been shown to localize to the nucleus of olfactory neurons in young animals [27] and in the glioblastoma cell line GM7 [28]. Olfactory neurons that had ENO2 localized to the nucleus, were from young animals, while in adults, ENO2 was exclusively localized to the cytoplasm. In the As^+3 ^and Cd^+2 ^transformed MCF-10A cells, ENO2 frequently localized to regions of the nucleus that had low levels of the nuclear stain To-PRO-3 iodide, a profile which might indicates a preferred localization to regions of euchromatin. Although ENO2 mRNA does not appear to have the alternate initiation site that codes for MBP-1, a protein which localizes to the nucleus, there is substantial homology between MBP-1 and the corresponding region in ENO2.

As detailed in the Introduction, immunostaining with ENO2, chromogranin A and synaptophysin have been used extensively to determine neuroendocrine differentiation in breast cancer. As a prognostic indicator, an immunostaining profile suggestive of neuroendocrine differentiation in breast cancer has been shown to have limited utility to enhance patient diagnosis or predict outcome [29-32]. However, there is evidence to suggest that ENO2 is altered in breast cancer even though its expression may not have independent prognostic value. Gene expression analysis has shown ENO2 mRNA to be elevated in breast cancer lymph node metastases compared to primary breast tumors [33] and ENO2 mRNA to be upregulated in the estrogen receptor positive subset of 36 invasive ductal breast carcinomas [34]. The findings in the present report that ENO2 expression in MCF-10A cells may be influenced by both methylation and histone modifications may provide a link between ENO2 expression in the breast epithelial cell and alterations in epigenetic gene regulation by the environmental pollutants, arsenic and cadmium. The earlier report documenting the malignant transformation of MCF-10A cells by Cd^+2 ^strengthens the evidence for a role for Cd^+2 ^in the development of breast cancer and also provides an extensive literature review on the available evidence linking breast cancer with exposure to Cd^+2 ^[24]. The evidence for a role of As^+3 ^in the etiology of breast cancer is far more limited with a major consideration being its ability, similar to Cd^+2^, to function as an endocrine disruptor [35,36]. Thus, the present study shows that acute and chronic exposure of MCF-10A cells to both As^+3 ^and Cd^+2 ^induces the expression of ENO2 and that the effects of As^+3 ^and Cd^+2 ^on ENO2 expression may be at the level of epigenetic modification.

## Conclusions

This study shows that the expression of the ENO2 gene in the breast epithelial cell can be induced by acute exposure to either As^+3 ^or Cd^+2 ^as well as by chronic exposure sufficient to allow colony formation of the cells in soft agar. Evidence is presented that control of ENO2 gene expression is controlled by methylation and histone modification.

## Methods

### Immunostaining for ENO1 and 2 in normal breast

Tissue sections for the immunohistochemical analysis of ENO1 and 2 expression in human breast were obtained from archival paraffin blocks that originated from previously completed patient diagnostic procedures. These archival specimens contained no patient identifiers and use was approved by the University of North Dakota Internal Review Board. Tissues were routinely fixed in 10% neutral buffered formalin for 16-18 hours. All tissues were transferred to 70% ethanol and dehydrated in 100% ethanol. Dehydrated tissues were cleared in xylene, infiltrated, and embedded in paraffin. Tissue sections were cut at 3-5 μm for use in immunohistochemical protocols. Prior to immunostaining, sections were immersed in preheated citrate buffer pH 6.0 and heated in a steamer for 20 minutes. The sections were allowed to cool to room temperature and immersed into Tris buffered saline with Tween 20 (Dako, Carpinteria, CA) for 5 minutes. The primary antibody for the localization of ENO1 (Abcam Inc., Cambridge, MA, Cat # Ab54979) was used at a 1:400 dilution and for ENO2 (Abcam Inc., Cambridge, MA, Cat # Ab54979) was used at a 1:250 dilution. Liquid diaminobenzidine was used for visualization. Slides were rinsed in distilled water, dehydrated in graded ethanol, cleared in xylene, and coverslipped.

### Cell culture

The MCF-10A cell line was obtained from the American Type Culture Collection and grown in a 1:1 mixture of Ham's F-12 medium and DMEM supplemented with 5% (v/v) fetal calf serum, 10 μg/ml insulin, 0.5 μg/ml hydrocortisone, 20 ng/ml epidermal growth factor, and 0.1 μg/ml cholera toxin. The cells were fed fresh growth medium every 3 days, and at confluence (normally 6-12 days post subculture), the cells were subcultured at a 1:10 ratio using trypsin-EDTA (0.25%, 1 mM). Preliminary experiments were performed to determine the approximate concentrations of Cd^+2 ^and As^+3 ^that would be at or below that resulting in MCF-10A cell toxicity over an acute time course of exposure. From this preliminary determination, 3 concentrations of CdCl_2 _(2, 4 and 6 μM) and 3 concentrations of NaAsO_2 _were chosen for a short-term exposure of 48 hrs. Cell viability and growth curves were determined by measuring the capacity of the cells to reduce MTT (3-(4,5-dimethylthiazol-2-yl)-2,5-diphenyltetrazolium bromide) to formazan [37]. Triplicate cultures were analyzed for each time point and concentration.

### Cadmium- and arsenite-induced transformation of MCF-10A cells

The protocol used to transform the MCF-10A cell line with Cd^+2 ^and As^+3 ^has been detailed previously for the human bladder UROtsa cell line [17]. An identical protocol starting with the parental MCF-10A cell line was used in the present study. Briefly, cultures of MCF-10A cells were grown to confluency in 25 cm^2 ^cell culture flasks and when confluent, each of the flasks were fed fresh growth media containing 1 μM As^+3 ^(NaAsO_2_, Fluka #71287) or 1 μM Cd^+2 ^(CdCl_2_, Sigma, St. Louis, MO). Following addition of the Cd^+2 ^and As^+3^, the cultures were thereafter fed fresh growth media every three days that contained As^+3 ^or Cd^+2^. The cultures were observed immediately before and 24 hrs after each feeding by light microscopy and subcultured at confluence.

The cultures were tested every 4 to 6 serial passages for their ability to form colonies in soft agar using a slight modification of the procedure described by San and coworkers [17,38]. Briefly, 60 mm diameter dishes were prepared with a 5 ml underlay of 0.5% agar in DMEM containing 5% fetal calf serum. On top of the under layer was placed 2 × 10^4 ^cells in 1.5 ml of 0.25% agar in DMEM containing 5% fetal calf serum. The dishes were incubated at 37°C in a 5% CO_2_: 95% air atmosphere inside humidified plastic containers to prevent evaporation. Cultures were examined microscopically 24 hrs after plating to confirm an absence of large clumps of cells and thereafter at 7, 14 and 21 days after plating. The respective cultures that showed colony formation in soft agar along with the MCF-10A parent cell line, were each inoculated subcutaneously (s.c.) at a dose of 1 × 10^6 ^cells in the dorsal thoracic midline of 5 nude (NCr-nu/nu) mice. Tumor formation and growth were assessed weekly. All mice were sacrificed 9 months following injection. All experimental procedures with the use of mice were approved by the University of North Dakota Institutional Animal Care and Use Committee and conform to the National Research Council's Guide for the Care and Used of Laboratory Animals. Areas where the cells were injected were paraffin-embedded, sectioned, stained with Hematoxylin and Eosin (H&E), and analyzed by light microscopy.

### Expression of ENO1, ENO2 and ENO3 mRNA and protein in the MCF-10A and MCF-10A derived cell lines

The procedures used for the preparation of total RNA and protein from cultured cells has been described previously by the laboratory [17,39]. The expression of ENO1, ENO2 and ENO3 mRNA was determined using real time RT-PCR and ENO1, 2 and 3 specific primers obtained from Qiagen (Valencia, CA). The levels of ENO1, 2 and 3 mRNA was determined relative to the MCF-10A cells using ENO-1, 2 and 3 standards to generate a standard curve. The expression levels of ENO-1, 2 and 3 were normalized to β-actin. The results are expressed in copy number/1000 β-actin transcripts and plotted as % change. The expression of ENO1 and 2 protein was determined by western blotting using 20 μg of total cellular protein on a 12.5% SDS-polyacrylamide gel using the above ENO1 and 2 antibodies at 1:100,000 and 1:2,000 dilutions, respectively. Anti-mouse IgG HRP-linked Ab was used to detect ENO-1 and anti-rabbit IgG, HRP-linked Ab used to detect ENO-2. The biotinylated molecular weight marker was detected with anti-biotin-HRP-linked antibody. The blots were visualized using the phototope-HRP western blot detection system.

### Immunolocalization of ENO1 and ENO2 in the MCF-10A and MCF-10A-derived cell lines

The cell lines were grown in 24 well plates containing 12 mm glass coverslips at 37°C, 5% CO_2_. Cells at a nearly confluent density were then fixed and stained according to published protocols [18,19]. Briefly, cells were fixed in 3.7% buffered, methanol-free formaldehyde (Polysciences, Inc, Warrington, PA) for 15-20 minutes at room temperature. Coverslips were then quenched of free aldehyde with 0.1 M NH_4_Cl for 15 minutes, followed by permeabilization with 0.1% Triton-X100 for 10 minutes. Cells were stained for ENO1 (primary and secondary antibodies) followed by staining for ENO2. Staining for ENO1 or ENO2 was carried out by incubation for 45-60 minutes at 37°C with 0.5 μg/ml of mouse anti-ENO1 or a 1:100 dilution of rabbit anti-ENO2 antibody. Primary antibodies were detected by incubating cells with 2.7 μg/ml of Alexa-Fluor 594 goat anti-mouse IgG or 2.7 μg/ml of Alexa-Fluor 488 goat anti-rabbit antibody (Invitrogen, Carlsbad, CA) for 45-60 minutes at 37°C. Controls consisted of coverslips treated with the appropriate secondary antibody(ies) only, staining for ENO1 or ENO2 alone with appropriate mouse or rabbit secondary antibodies as well as with in-appropriate rabbit or mouse secondary antibodies, respectively. All controls stained appropriately and negative controls had virtually no staining when photographed under the same settings that were used for experimental cells. For experiments determining the localization of ENO2 to the nucleus, ENO2 staining was carried out as indicated above followed by staining with a 5 μM solution of To-PRO-3 iodide (Invitrogen) for 30 minutes at 37°C. All coverslips were mounted in ProLong Gold anti-fade reagent with DAPI (Invitrogen) for nuclear counter staining. Cells were observed and images captured using a Zeiss LSM 510 Meta Confocal Microscope with LSM 510 software (Carl Zeiss MicroImaging Inc). Images were obtained by capturing z-slices at a depth of 0.46 μm. DAPI images of the same fields were captured by epifluorescence.

### Treatment of MCF-10A cells with 5-Azacytidine and histone deacetylase inhibitor MS-275

The MCF-10A cells were seeded at a ratio of 1:10 and the next day they were exposed to 0.5 and 1.5 μM 5-azacytidine (AZC) or the histone deacetylase inhibitor MS-275 at 1.0, 3.0, and 10 μM for three days till the cells reached confluency as described previously [20]. Cells were then harvested for the determination of ENO2 mRNA expression.

## Competing interests

The authors declare that they have no competing interests.

## Authors' contributions

The current study was the Ph.D. dissertation project for MS, and SG participated as the Graduate Student Dissertation Director and assisted with data analysis. SS supervised cell culture and western analysis, JD supervised immunofluorescent localization, and XZ supervised and help perform immunohistochemistry. MAS was responsible for informed consent, patient samples, and was the originator of the project. CB performed the chronic exposure of the MCF-10A cells to As^+3 ^and Cd^+2^, and CA was an undergraduate student who made the initial discovery of ENO2 overexpression in transformed cells. DAS wrote the paper with MS, designed the study, and was involved in overall data interpretation. All authors have read and approved the final manuscript.
